# A comparative proteomics study of a synovial cell line stimulated with TNF‐α

**DOI:** 10.1002/2211-5463.12049

**Published:** 2016-03-31

**Authors:** Seiji Shibasaki, Miki Karasaki, Shunsuke Aburaya, Hironobu Morisaka, Yumiko Takeda, Wataru Aoki, Sachie Kitano, Masayasu Kitano, Mitsuyoshi Ueda, Hajime Sano, Tsuyoshi Iwasaki

**Affiliations:** ^1^ General Education CenterHyogo University of Health SciencesKobeJapan; ^2^ Division of Applied Life ScienceGraduate School of AgricultureKyoto UniversityJapan; ^3^ Division of RheumatologyDepartment of Internal MedicineHyogo College of MedicineNishinomiyaJapan; ^4^ Division of PharmacotherapyDepartment of PharmacySchool of PharmacyHyogo University of Health SciencesKobeJapan

**Keywords:** apoptosis, comparative proteomics, gene ontology analysis, rheumatoid arthritis, synovial cell line, TNF‐α

## Abstract

To elucidate the pathogenesis of rheumatoid arthritis (RA), we used proteomic analysis to determine the protein profile in a synovial cell line, MH7A, established from patients with RA. Proteins were extracted from MH7A cells that were or were not stimulated with tumor necrosis factor‐α (TNF‐α), and then analyzed on a liquid chromatography/mass spectrometry system equipped with a unique long monolithic silica capillary. On the basis of the results of this proteomic analysis, we identified 2650 proteins from untreated MH7A cells and 2688 proteins from MH7A cells stimulated with TNF‐α. Next, we selected 269 differentially produced proteins that were detected only under TNF‐α stimulation, and classified these proteins by performing gene ontology analysis by using DAVID as a functional annotation tool. In TNF‐α‐stimulated MH7A cells, we observed substantial production of plasminogen‐activator inhibitor 2 and apoptosis‐regulating proteins such as BH3‐interacting domain death agonist, autophagy protein 5, apolipoprotein E, and caspase‐3. These results indicate that the upregulation of plasminogen‐activator inhibitor 2 and apoptosis‐regulating proteins in synovial cells in response to TNF‐α stimulation might represent a predominant factor that contributes to the pathogenesis of RA.

**Abbreviations**
FLS: fibroblast‐like synoviocytes
GO: gene ontology
IL‐6: interleukin‐6
LC: liquid chromatography
MS: mass spectrometry
PAI: plasminogen‐activator inhibitor
RA: rheumatoid arthritis
TNF‐α: tumor necrosis factor‐α

Rheumatoid arthritis (RA) is characterized by a proliferation of synovial cells that leads to joint destruction 1. The pathogenesis of RA comprises a preclinical stage involving the generation of autoantibodies, an initiation stage in which synovial inflammation emerges, and a clinical stage dominated by synovial inflammation and joint destruction 2. In RA pathogenesis, a central role is played by cytokines derived from macrophages and fibroblast‐like synoviocytes (FLS), such as tumor necrosis factor‐α (TNF‐α) and interleukin‐6 (IL‐6) 3. RA FLS have been described as transformed cells and they share morphologic features with tumor cells, such as resistance to apoptosis, potentially due to somatic mutations in p53 4, and RA FLS display and retain an invasive capacity against articular cartilage 5. Although FLS are the major synovial producers of IL‐6, a key pathogenic cytokine 6, 7, macrophages are likely the major source of synovial TNF‐α, a critical driver of the synovial inflammation that occurs in RA 4, 7. However, macrophages produce TNF‐α only transiently 8 and FLS are the major responders to TNF‐α, which suggest that TNF‐α‐stimulated FLS are crucial for the pathogenesis of RA 9. Therefore, we sought to identify the functional protein profile in FLS stimulated by TNF‐α.

Proteome analysis is an indispensable technique for comprehensively analyzing differences in protein production. One of the critical purposes of clinical proteome analysis is the identification of biomarkers for disease diagnosis and therapeutic intervention: the biomarkers are identified by analyzing the proteome profiles of disease states and other physiologic states 10. Recent high‐throughput and sensitive mass spectroscopy (MS) based on proteome‐analysis technology has provided a novel methodology for investigating the physiology and pathophysiology of numerous medicinal samples. In the case of protein separation coupled with MS analysis, two‐dimensional polyacrylamide gel electrophoresis (2D‐PAGE) has been widely used, but the low‐throughput performance of 2D‐PAGE could become a disadvantage in analyses that require the examination of several samples. The shotgun method is an alternative approach used in proteome analysis that involves liquid chromatography (LC) and tandem MS 11. Previously, we constructed a system that exhibits ultraperformance in chromatographic separation achieved with a long monolithic silica capillary column; this system has enabled, for example, successful one‐shot identification of 1658 proteins produced in *Mesorhizobium loti* in nodule or free‐living conditions 12. Thus, we adapted this nano LC‐MS/MS proteome‐analysis system for investigation into the mechanism of RA onset.

In this study, we stimulated a transformed FLS cell line, MH7A 13, 14, 15, 16, with TNF‐α and analyzed the intracellular protein profile by using an LC‐MS/MS system equipped with a 500‐cm‐long monolithic silica capillary column. We observed substantial production of plasminogen‐activator inhibitor (PAI) 2 and several apoptosis‐regulating proteins in TNF‐α‐stimulated MH7A cells. Thus, TNF‐α‐stimulated upregulation of PAI‐2 and apoptosis‐regulating proteins in synovial cells might play a crucial role in the pathogenesis of RA.

## Materials and methods

### Cell line

The human MH7A synovial cell line (Riken, Saitama, Japan), which originated from intra‐articular soft tissue of the knee joints of an RA patient, was established by transfecting cells with SV40 T antigen 13, 14, 15, 16. MH7A cells were cultured in Pure Coat 6‐well plates (BD, Franklin Lakes, NJ, USA) with RPMI 1640 medium (Sigma, St. Louis, MO, USA) containing 10% heat‐inactivated fetal bovine serum (Whittaker, Walkersville, MD, USA) and 100 U·mL^−1^ penicillin and 100 μg·mL^−1^ streptomycin (Invitrogen, Carlsbad, CA, USA). The culture plates were incubated for 24 h at 37 °C in an atmosphere of 5% CO_2_ in air. Subsequently, the plates were incubated for another 24 h in the presence or absence of 100 ng·mL^−1^ TNF‐α.

### Protein preparation

Culture medium was aspirated from culture plates and the cells were washed twice with ice‐cold PBS, after which ice‐cold PBS was added to the wells and cells were removed from the plates by using a cell‐scraper. These collected cells were transferred to conical tubes and total proteins were extracted using a Qproteome Mammalian Protein Prep Kit (Qiagen, Hilden, Germany).

### Trypsin digestion

We mixed 1 mL of the protein supernatant with 135 μL of 200 mm triethylammonium bicarbonate (TEAB), 165 μL of distilled water, and 15 μL of 200 mm tris‐(2‐carboxyethyl) phosphine (Thermo Fisher Scientific, Waltham, MA, USA). The mixture was incubated at 55 °C for 1 h, after which 15 μL of 375 mm iodoacetamide was added to the solution and incubated for 30 min. Next, the reactants were mixed with 3 mL of ice‐cold acetone and incubated at −20 °C for 2 h to precipitate proteins. Last, the precipitated proteins were resuspended in 100 μL of TEAB and mixed with 2 μL of 1 μg·μL^−1^ sequencing‐grade modified trypsin (Promega, Madison, WI, USA) at 37 °C overnight.

### LC‐MS/MS analysis

Proteome analyses were performed using an LC‐MS system (LC, UltiMate 3000 RSLCnano System, and MS, LTQ Velos Orbitrap mass spectrometer; Thermo Fisher Scientific) that was equipped with a long monolithic silica capillary column (500 cm long, 0.1 mm ID; Kyoto Monotech, Kyoto, Japan). Tryptic digests (5 μL) were injected and separated through reversed‐phase chromatography at a flow rate of 500 nL·min^−1^; the gradient was produced by changing the mixing ratio of these two eluents: A, 0.1% (v/v) formic acid, and B, 80% (v/v) acetonitrile containing 0.1% (v/v) formic acid. The gradient was started with 5% B, increased to 45% B for 600 min, further increased to 95% B to wash the column, and then returned to the initial condition and held for re‐equilibration. The separated analytes were detected on a mass spectrometer with a full scan range of 350–1500 *m*/*z*. For data‐dependent acquisition, the method was set to automatically analyze the top‐10 most intense ions observed in the MS scan. An ESI voltage of 2.3 kV was applied directly to the LC buffer distal to the chromatography column by using a MicroTee. The temperature of the ion transfer tube was set to 280 °C.

### Data analysis

All samples were subjected to triplicate LC‐MS/MS analysis, and the combined spectrometry data were used for protein identification. Proteins were identified by using MASCOT (Matrix Science, London, UK) against the assembly 21 protein database at SwissProt (2002–2015 UniProt Consortium, EMBL‐EBI) that contains 20210 sequences, with a precursor mass tolerance of 20 ppm, fragment tolerance of 0.8 Da, and strict specificity allowing for up to one missed cleavage. For trypsin digestion, carbamidomethylation of cysteine was set as a fixed modification, and oxidation of methionine was set as a dynamic modification. The data were then filtered at a *q*‐value ≤ 0.01 corresponding to 1% false‐discovery rate on a spectral level. Triplicate analyses were performed for each sample of three biological replicates. The protein identification threshold was as follows; proteins identified using more than one unique peptide and three peptides; if not, proteins identified using a single unique peptide for each biological replicate. For gene ontology (GO) analysis, the DAVID annotation tool was used. A significant enrichment score means that the number of identified proteins belonging to their annotation term is significantly enriched comparing to the number of genes belonging to that. For comparing the protein amount of each gene, exponentially modified protein abundance index (emPAI) 17 was calculated with MACOT. An emPAI offers approximate, semiquantitative value. It is developed by Ishihama *et al*. 17 and emPAI values were calculated from number of identified peptides per protein and identifiable peptides per protein.

## Results

### Screening for differentially produced proteins

**Figure 1:**
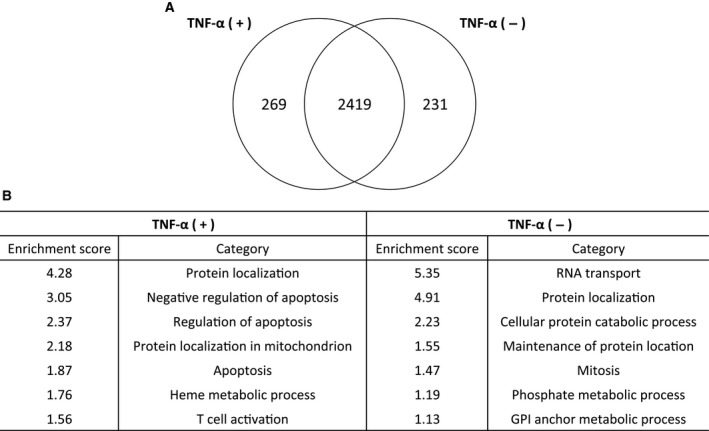
Summary of protein identification in MH7A cells. (A) Venn diagram of identified proteins; (B) gene ontology analysis of proteins identified with/without TNF‐α stimulation.

We conducted three independent experiments by using cultured MH7A cells that were untreated or stimulated with TNF‐α. On the basis of the results of the proteome analysis, we identified 2650 proteins from untreated MH7A cells and 2688 proteins from TNF‐α‐stimulated MH7A cells, and then selected 269 differentially produced proteins that were detected under TNF‐α stimulation (Fig. 1).

### GO analysis of differentially produced proteins

The proteins that were differentially produced following stimulation with TNF‐α were classified according to GO analysis performed by using DAVID as the functional annotation tool. The enrichment scores obtained for protein localization, negative regulation of apoptosis, regulation of apoptosis, protein localization in mitochondria, apoptosis, heme metabolic process, and T‐cell activation were 4.28, 3.05, 2.37, 2.18, 1.87, 1.76, and 1.56, respectively (Fig. 1).

### Plasminogen‐activator inhibitor 2 and apoptosis‐regulating proteins

Among the differentially produced proteins analyzed, we detected TNF‐α signaling proteins such as nuclear factor‐kappa‐B (NF‐κB) and AP‐1 complex subunit σ1A, and synovial cell growth proteins such as transforming growth factor‐β1 and IL‐6 receptor‐β. We also observed substantial production of PAI‐2 and apoptosis‐regulating proteins such as BH3‐interacting domain death agonist (BID), autophagy protein 5 (ATG5), apolipoprotein E (ApoE), and caspase‐3 in TNF‐α‐stimulated MH7A cells (Table 1).

**Table 1:** Exponentially modified protein abundance index (emPAI) value of identified proteins. emPAI is defined as, emPAI = 10^PAI^−1. PAI = the number of identified peptides/the number of identifiable peptides

Accession number	Average emPAI	Description
Protein localization		
P62745	0.58	Rho‐related GTP‐binding protein RhoB
Q9Y5J9	0.36	Mitochondrial import inner membrane translocase subunit Tim8 B
O60831	0.3	PRA1 family protein 2
Q9BRG1	0.29	Vacuolar protein‐sorting‐associated protein 25
P62072	0.22	Mitochondrial import inner membrane translocase subunit Tim10
Q9NQY0	0.2	Bridging integrator 3
P61966	0.18	AP‐1 complex subunit sigma‐1A
Q13637	0.18	Ras‐related protein Rab‐32
O00560	0.17	Syntenin‐1
Negative regulation of apoptosis		
P05120	1.21	Plasminogen activator inhibitor 2 (PAI‐2)
O60936	0.24	Nucleolar protein 3
P37840	0.24	Alpha‐synuclein
P16989	0.16	Y‐box‐binding protein 3
Q9UEE9	0.14	Craniofacial development protein 1
P42574	0.1	Caspase‐3
Q9H1Y0	0.1	Autophagy protein 5 (ATG5)
P02649	0.09	Apolipoprotein E
Q86SJ2	0.06	Amphoterin‐induced protein 2
P19838	0.05	Nuclear factor NF‐kappa‐B p105 subunit
Regulation of apoptosis		
P05120	1.21	Plasminogen activator inhibitor 2 (PAI‐2)
O60936	0.24	Nucleolar protein 3
P37840	0.24	Alpha‐synuclein
P16989	0.16	Y‐box‐binding protein 3
P31483	0.16	Nucleolysin TIA‐1 isoform p40
P55957	0.15	BH3‐interacting domain death agonist (BID)
Q9UEE9	0.14	Craniofacial development protein 1
P42574	0.1	Caspase‐3
Q6SZW1	0.1	Sterile alpha and TIR motif‐containing protein 1
Q9H1Y0	0.1	Autophagy protein 5 (ATG5)
Protein localization of mitochondrium		
Q9Y5J9	0.36	Mitochondrial import inner membrane translocase subunit Tim8 B
P62072	0.22	Mitochondrial import inner membrane translocase subunit Tim10
P55957	0.15	BH3‐interacting domain death agonist (BID)
P01137	0.07	Transforming growth factor beta‐1
Q96BW9	0.06	Mitochondrial translocator assembly and maintenance protein 41 homolog
P30536	0	Translocator protein
Apoptosis		
P62745	0.58	Rho‐related GTP‐binding protein RhoB
O60936	0.24	Nucleolar protein 3
Q9NP84	0.24	Tumor necrosis factor receptor superfamily member 12A
Q8IXM3	0.22	39S ribosomal protein L41, mitochondrial
P31483	0.16	Nucleolysin TIA‐1 isoform p40
P55957	0.15	BH3‐interacting domain death agonist (BID)
P42574	0.1	Caspase‐3
Q9H1Y0	0.1	Autophagy protein 5 (ATG5)
Q9NXR7	0.08	BRCA1‐A complex subunit BRE
O43464	0.07	Serine protease HTRA2, mitochondrial
Heme metabolic process		
Q96EY8	0.16	Cob(I)yrinic acid a,c‐diamide adenosyltransferase, mitochondrial
P13716	0.12	Delta‐aminolevulinic acid dehydratase
P13196	0.09	5‐aminolevulinate synthase, nonspecific, mitochondrial
Q9NQX3	0.04	Gephyrin
P30536	0	Translocator protein
T‐cell activation		
Q9BQ51	0.11	Programmed cell death 1 ligand 2
P42574	0.1	Caspase‐3
P01137	0.07	Transforming growth factor beta‐1
Q6QNY0	0.05	Biogenesis of lysosome‐related organelles complex 1 subunit 3
P40189	0.02	Interleukin‐6 receptor subunit beta
P04626	0	Receptor tyrosine‐protein kinase erbB‐2

## Discussion

**Figure 2:**
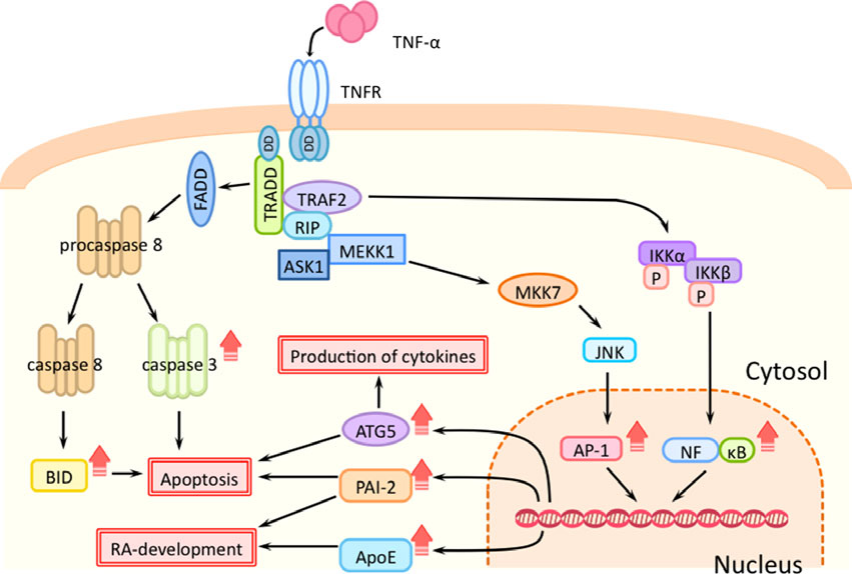
Intracellular signaling by TNF‐α. An up arrow means that molecules were detected in this study.

In TNF‐α‐stimulated MH7A cells, we detected NF‐κB and AP‐1 complex subunit σ1A. This suggested that TNF‐α‐induced intracellular signaling (Fig. 2) was operating in the MH7A cells used in this experiment 18.

In MH7A cells stimulated with TNF‐α, PAI‐2 was also produced. Abnormalities of the synovial lining layer might represent an extremely early feature of synovitis in RA. Even in an early clinical stage, the surface of the synovial lining layer is frequently covered with fibrin deposits generated from the activation of the fibrinolytic system by synovial fluid. The synovial lining layer is completely replaced by a fibrin cap, and in highly inflamed tissues, this fibrin can extend into the sublining layers.

Proteolytic digestion of the fibrin clot is mediated by plasminogen activation. The plasminogen activation system has been widely suggested to play a crucial pathogenic role in destructive joint disease 19, 20, 21. Busso *et al*. 22 reported that the concentrations of urokinase‐type plasminogen activator (u‐PA), PAI‐1, PAI‐2, and u‐PA receptor were markedly higher in RA patients than in osteoarthritis patients. The u‐PA, u‐PA receptor, and PAI proteins produced in increased amounts were distributed mainly in the synovial lining area of proliferative and invasively growing synovial tissue in RA patients 22. Our detection of substantial PAI‐2 production in MH7A cells after TNF‐α stimulation potentially supports these previous observations (Table 1).

We detected markedly increased production of apoptosis‐regulating proteins such as ApoE, ATG5, and BID in TNF‐α‐stimulated MH7A cells (Table 1). ApoE might play a role in RA. A recent study demonstrated that ApoE‐deficient mice were resistant to the development of collagen‐induced arthritis 23. Synovial fluid samples collected from the inflamed joints of RA patients contain several citrullinated proteins. Citrullinated ApoE is a newly identified antigen in RA synovial fluid, and only a limited number of the protein's citrullinated epitopes are targeted by the immune system in RA 24. Thus, the increased ApoE production in TNF‐α‐stimulated MH7A cells observed here suggests that the induction of citrullinated ApoE and autoimmune reactions could be involved in RA development (Fig. 2).

Autophagy is a highly conserved lysosome‐mediated catabolic process and a homeostatic process that degrades unnecessary or dysfunctional cellular organelles and recycles nutrients. ATG5 is a critical protein required for autophagy at the stage of autophagosome‐precursor synthesis. In addition to regulating autophagosome formation, ATG5 might play an important role in apoptosis 25, 26, 27. Moreover, ATG5 regulates the production of inflammatory cytokines, the functions of antigen‐presenting cells, and the clearance of apoptotic cells, which indicates that ATG5 might contribute to the pathogenesis of RA 28, 29, 30, 31. Our finding that ATG5 production was increased in TNF‐α‐stimulated MH7A cells suggests that apoptosis induction and immune‐function regulation by this protein could underlie the development of RA (Fig. 2).

BID is a pro‐apoptotic BH3‐only Bcl‐2 homolog that is involved in “extrinsic” apoptosis: BID is activated by cleavage only in response to cell‐surface receptors that transmit apoptotic signals initiated by the specific ligand granzyme B produced by cytotoxic cells 32. Peripheral blood neutrophils that were dying spontaneously as a result of apoptosis (i.e., through “intrinsic” apoptosis) showed no BID cleavage despite efficient caspase‐3 activation. By contrast, prominent BID cleavage was detected in neutrophils that were exposed to granzyme B/perforin or in neutrophils in which death receptor was activated (with anti‐Fas or TNF‐α/cycloheximide). In RA synovial fluid, BID cleavage was closely associated with the cleavage of caspase‐3 33. The increased BID production in TNF‐α‐stimulated MH7A cells observed in this study suggests that RA development might involve BID activation‐dependent induction of apoptosis (Fig. 2).

Previously, TNF‐α was demonstrated to induce prolonged activation of NF‐κB signaling and transcription of mRNAs encoding IL‐6 and chemokines and chemokine receptors such as CXCL8/IL‐8 and CCL5/RANTES 34. A comparative proteomics study previously showed that proteins related to vasculature development were upregulated in RA FLS as compared with the corresponding levels in normal FLS 35. However, this study has demonstrated for the first time that the plasminogen activation system is upregulated in synovial cells after TNF‐α stimulation (Fig. 2).

In conclusion, we detected substantial production of PAI‐2 and several apoptosis‐regulating proteins in TNF‐α‐stimulated MH7A cells. Our results indicate that these upregulated proteins might contribute to the pathogenesis of RA.

## Author contributions

S.S., M.U., and T.I. conceived and designed the project, M.K., S.A., H.M., Y.T., S.K., and M.K. acquired the data, S.A., H.S., and W.A. analyzed and interpreted the data, S.S., M.U., and T.I. wrote the paper.
